# Effects of Abscisic Acid, Gibberellin, Ethylene and Their Interactions on Production of Phenolic Acids in *Salvia miltiorrhiza* Bunge Hairy Roots

**DOI:** 10.1371/journal.pone.0072806

**Published:** 2013-09-02

**Authors:** Zongsuo Liang, Yini Ma, Tao Xu, Beimi Cui, Yan Liu, Zhixin Guo, Dongfeng Yang

**Affiliations:** 1 College of Life Science of Zhejiang Sci-Tech University, Hangzhou, China; 2 College of Life Science of Northwest A&F University, Yangling, China; 3 Tianjin Tasly Modern TCM Resources Co., Ltd., Tianjin, China; 4 Tasly Institute, Tianjin, China; University of Wisconsin - Madison, United States of America

## Abstract

*Salvia miltiorrhiza* is one of the most important traditional Chinese medicinal plants because of its excellent performance in treating coronary heart disease. Phenolic acids mainly including caffeic acid, rosmarinic acid and salvianolic acid B are a group of active ingredients in *S. miltiorrhiza*. Abscisic acid (ABA), gibberellin (GA) and ethylene are three important phytohormones. In this study, effects of the three phytohormones and their interactions on phenolic production in *S.* *miltiorrhiza* hairy roots were investigated. The results showed that ABA, GA and ethylene were all effective to induce production of phenolic acids and increase activities of PAL and TAT in *S. miltiorrhiza* hairy roots. Effects of phytohormones were reversed by their biosynthetic inhibitors. Antagonistic actions between the three phytohormones played important roles in the biosynthesis of phenolic acids. GA signaling is necessary for ABA and ethylene-induced phenolic production. Yet, ABA and ethylene signaling is probably not necessary for GA3-induced phenolic production. The complex interactions of phytohormones help us reveal regulation mechanism of secondary metabolism and scale-up production of active ingredients in plants.

## Introduction

**Figure 1:**
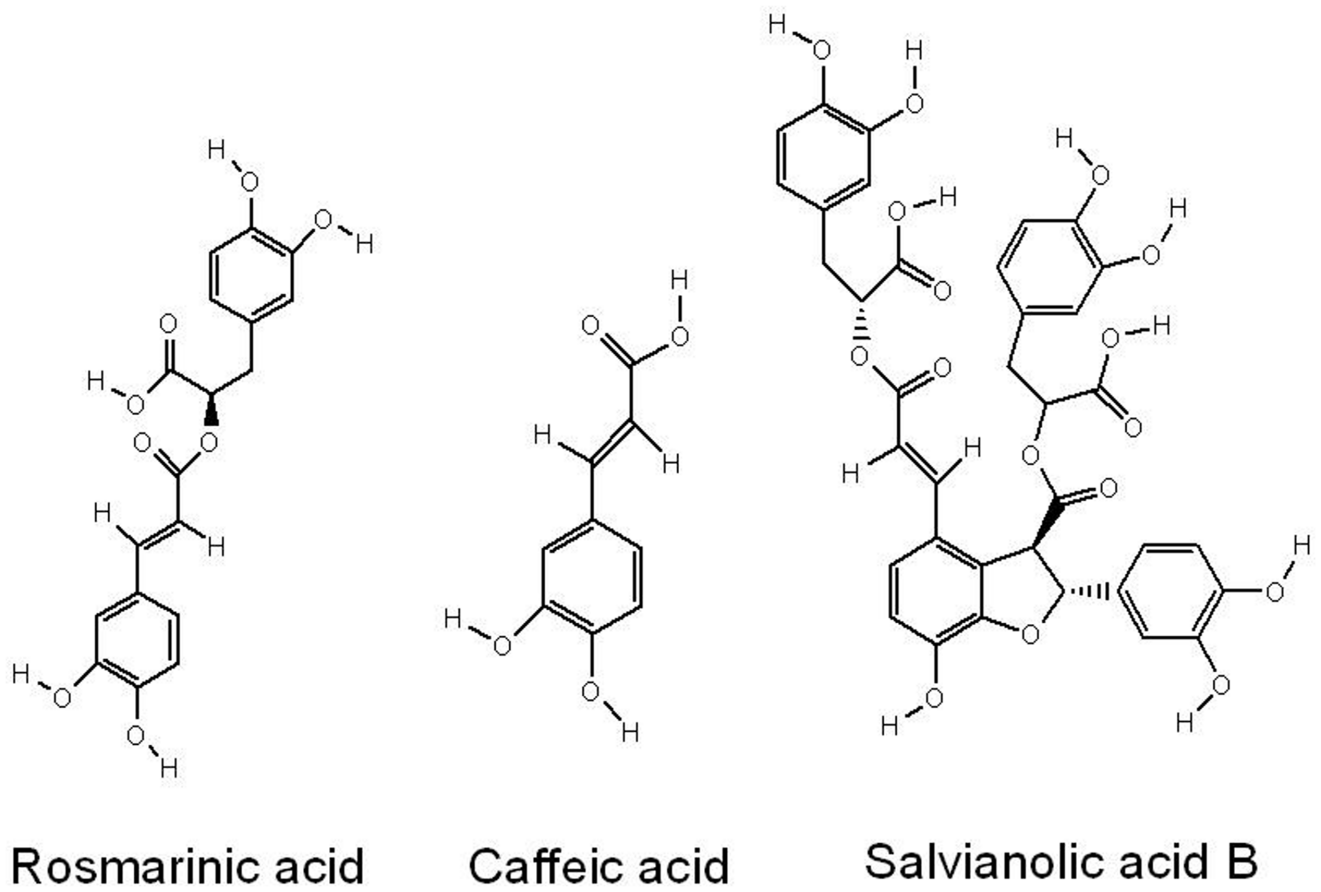
Chemical structures of salvianolic acid B, rosmarinic acid and caffeic acid.

*Salvia miltiorrhiza* Bunge (Danshen in Chinese) is a very useful traditional Chinese medicine in treatment of myocardial infraction, angina pectoris, heart diseases, stroke, alzheimer’s disease, cardiovascular and cerebrovascular diseases [1]. Demands of *Salvia miltiorrhiza* in China are more 4, 000, 000 kilograms, which needs 160 km^2^ cultivated land to produce. Improvement of *Salvia miltiorrhiza* quality or industrialization of active ingredients production is very important to save cultivated land. There are two major groups of active ingredients in *S. miltiorrhiza*, tanshinones and phenolic acids. These years, more and more attentions have been paid to phenolic acids in *S. miltiorrhiza* roots because of their excellent effects on heart disease [2]. Phenolic acids including salvianolic acid B (SAB), rosmarinic acid (RA) and caffeic acid (CA) (Fig. 1) in *S. miltiorrhiza* are biosynthesized via the phenylpropanoid and the tyrosine-derived pathways. Phenylalanine ammonia-lyase (PAL) and tyrosine aminotransferase (TAT) are two key enzymes involved in the biosynthesis of phenolic acids. Various elicitors have been investigated to stimulate phenolic production in *S. miltiorrhiza* hairy roots,including yeast extracts, Ag^+^, methyl jasmonate, salicylic acid and abscisic acid [3]. However, our knowledge about the regulation mechanism of phenolic biosynthesis in *S. miltiorrhiza* is far from complete.

Phytohormones, a group of crucial signal molecules, not only regulated all aspects of plant growth and development, but also were involved in plant secondary metabolism [4]. Many phytohormones have been used as efficient elicitors to stimulate production of plant secondary metabolites. ABA was defined as a stress plant hormone because of its rapid accumulation in response to stress. We found that accumulations of tanshinones and phenolic acids were significantly improved by ABA treatment [5], [6], [7], [8]. Ethylene was a phytohormone that regulated a wide range of plant processes. It has been reported that ethylene is effective to induce anthocyanin production in strawberry [9] and phenolic production in carrots [10]. Gibberellin (GA) was also well known as an effective elicitor for production of secondary metabolites. The previous work indicated that tanshinone production in *S. miltiorrhiza* hairy roots were significantly induced by gibberellic acid 3 (GA3) [11].

Interactions of phytohormone signalings in regulating plant development and metabolism have been widely reported [12], [13]. GA was regarded as an antagonist of ABA and ethylene. Effects of ABA on plants could be counteracted by applications of GA and ethylene [14], [15], [16], [17]. The antagonistic action between GA and ABA was an important factor regulating the developmental transition from embryogenesis to seed germination [14]. Ethylene could also partially inhibited the action of GA on seedlings of *Alaska pea* [18]. Although our previous work has shown that production of phenolic acids in *S. miltiorrhiza* hairy roots is stimulated by ABA, effects of ethylene and GA on phenolic production are still unknown. Interactions between the three phytohormones are still unclear. The goal of this study is to investigate effects of GA and ethylene on the accumulations of phenolic acids in *S. miltiorrhiza* hairy roots and reveal interactions between the three phytohormones.

## Materials and Methods

### Preparation for Phytohormones and Inhibitors

ABA (Wolsen, China), gibberellic acid 3 (GA3, Wolsen, China ), ethephon (Eth, Sigma, USA), and CoCl_2_ (ethylene biosynthesis inhibitor, Sigma, USA) were dissolved in distilled water. Paclobutrazol (GA biosynthetic inhibitor, Sigma, USA) and fluridone (ABA biosynthesis inhibitor, Augsburg, Germany) were dissolved in 70% ethanol. Finally, all the reagents were sterilized by filtering through a microfilter (0.2 µm) and stored at 4°C in a refrigerator prior to use.

### Hairy Root Culture and Treatment

The *S. miltiorrhiza* hairy roots were derived after the infection of *Agrobacterium rhizogenes* bacterium (ATCC15834). The 6,7-V medium was chosen as the basal medium with 20 g/L sucrose and pH was adjusted to 5.6–5.8 with NaOH. All experiments in this research were performed in suspension culture of *S. miltiorrhiza* hairy roots in a liquid medium of 6, 7-v. Hairy roots with 0.2 g fresh roots were cultivated in 250-ml shake flasks containing 50 ml of the hormone-free liquid basal medium on an orbital shaker at 110 rpm and 25°C in the dark.

Treatments of phytohormones and inhibitors were conducted on the 18^th^ day after inoculation of the hairy root cultures. Initially, four concentrations of GA3 (10, 50, 100 and 150 µM) and Eth (50, 100, 200 and 400 µM) were added into the culture medium, respectively. Afterwards, 100 µM GA3 and 50 µM Eth were chosen as the best concentrations. Then, three concentrations of paclobutrazol (26, 52 and 104 µM) and CoCl_2_ (50, 100 and 150 µM) were applied into the cultures. Concentrations of paclobutrazol at 52 µM and CoCl_2_ at 100 µM were finally selected. Concentrations of ABA (50 µM) and fluridone (34 µM) were chosen according to our previous study [8]. Finally, the selected concentrations of phytohormones and their inhibitors were employed to study their interactions on production of phenolic acids in *S. miltiorrhiza* hairy roots. The equal volume of distilled water was added to the hairy root cultures as the control.

Hairy roots were harvested from the culture medium by filtration on the 6^th^ day after treatments, blotted dry with paper towels (yielding the fresh weight, FW), and then dried at 45°C in an oven until constant weight (yielding the dry weight, DW). All treatments were performed in triplicate.

### Extraction and HPLC Analysis of Phenolic Acids

The dried hairy roots were comminuted to powder in a mortar and sieved through a 0.45-mm screen. The powder (0.1 g) was extracted ultrasonically with 2 ml methanol-water solution (7∶3) for 45 min. The extracts were centrifuged at 10,000 rpm for 15 min and filtered through a 0.45-µm Millipore filter. Then, contents of phenolic acids with HPLC was measured according to the method established in our lab [8].

### Statistical Analysis

All the data were analyzed using SPSS 16.0 for Windows. The data were initially compared by one way analysis of variance (ANOVA) and difference was detected using the Tukey test. The difference between treatments was considered to be statistically significant when *p* values ≤0.05. Figures were performed using GraphPad Prism 5 and means ± standard deviation (S.D.) (*n = 3*) were shown.

## Results

### Effects of Phytohormones and Inhibitors on Growth of *S.* *miltiorrhiza* Bunge Hairy Roots

**Figure 2:**
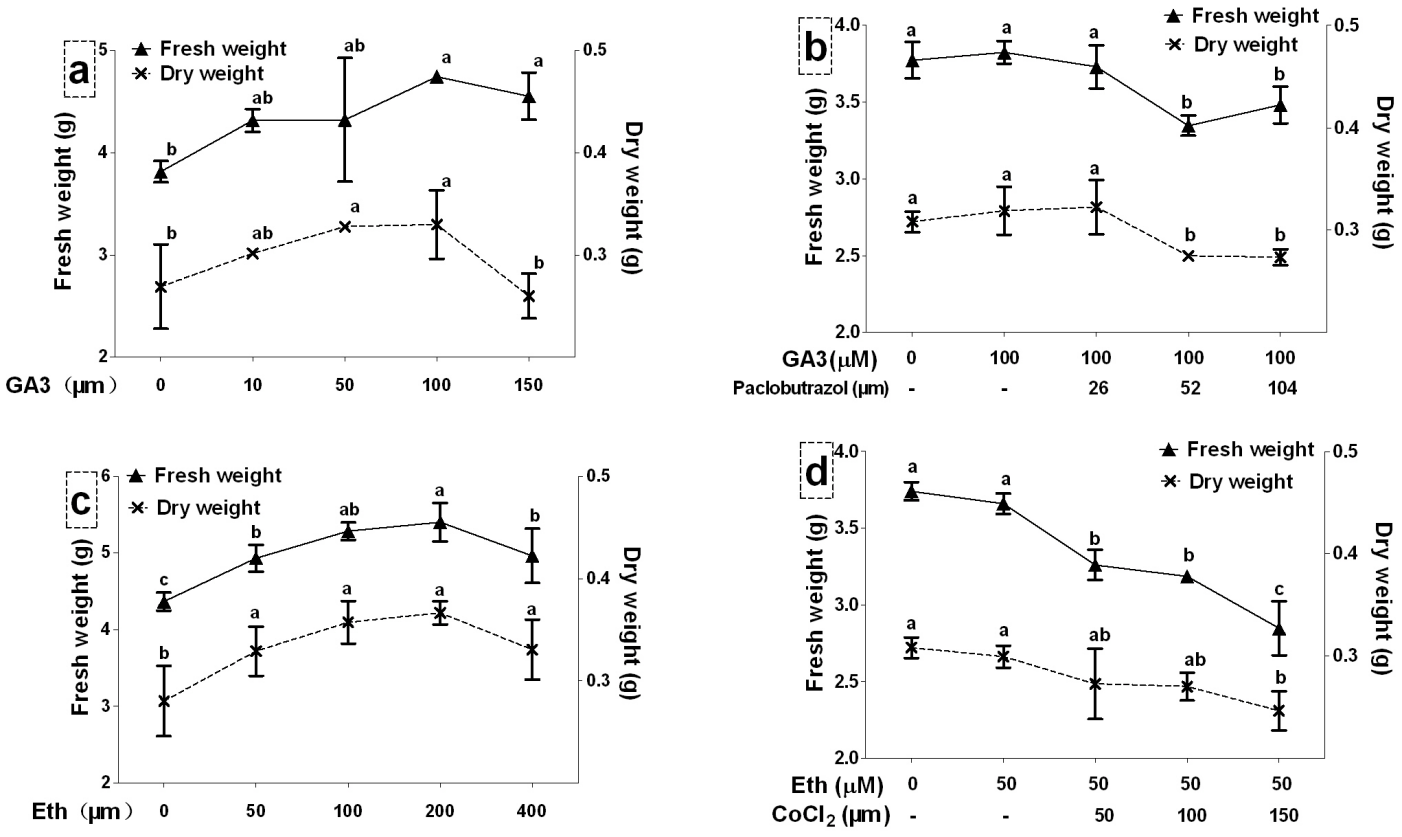
Effects of gibberellin acid and ethylene and their biosynthetic inhibitors on cell growth of *S. miltiorrhiza* hairy roots. GA3, pibberellin acid 3; Eth, ethephon. Different letters indicate significant difference at *p*≤0.05 using Tukey test test. Means ± standard deviation (S.D.) (*n = 3*) are shown.

Four concentrations of ABA, GA3 and Eth were separately added into the cultures to investigate their effects on *S. miltiorrhiza* hairy roots. Then, the inhibitors in combination with phytohormones were applied into the cultures to elucidate regulation mechanism of phenolic biosynthesis. The growth of *S. miltiorrhiza* hairy roots on day 6 after treatments was initially measured. Effects of ABA and fluridone on growth of *Salvia miltiorrhiza* hairy roots have been reported in our previous work [8]. Effects of GA3 on the growth of *Salvia miltiorrhiza* hairy roots were shown in Fig. 2a. The results showed that the fresh weight and dry weight were almost not affected by 10 µM GA3, while they were significantly increased by 100 µM GA3 (from 3.81 to 4.75 g FW and from 0.27 to 0.33 g DW). The fresh weight was almost not affected by 50 µM GA3, but significantly increased by 150 µM GA3. The dry weight was significantly increased by 50 µM GA3, but almost not affected by 150 µM GA3. A low concentration of paclobutrazol (26 µM) could not affect the growth of GA3-induced hairy roots, while the higher concentrations (52 and 104 µM) led to significant decreases as shown in Fig. 2b.

Treatments with four concentrations of Eth (50, 100, 200 and 400 µM) resulted in significant increases of the hairy root growth as shown in Fig. 2c. The highest fresh weight and dry weight were observed in 200 µM Eth treatment (to 5.40 g FW and 3.66 g DW, respectively). Treatments with CoCl_2_ (50, 100 and 150 µM) inhibited the growth of Eth-induced hairy roots (Fig. 2d). The higher the CoCl_2_ concentration was, the lower the hairy root weight was. When compared to 100 µM Eth treatment, the fresh weight and dry weight in 100 µM Eth +150 µM CoCl_2_ treatment were decreased by 22.2 and 17.7%.

### Effects of Phytohormones on Accumulation of Phenolic Acids in *S. miltiorrhiza* Hairy Roots

**Figure 3:**
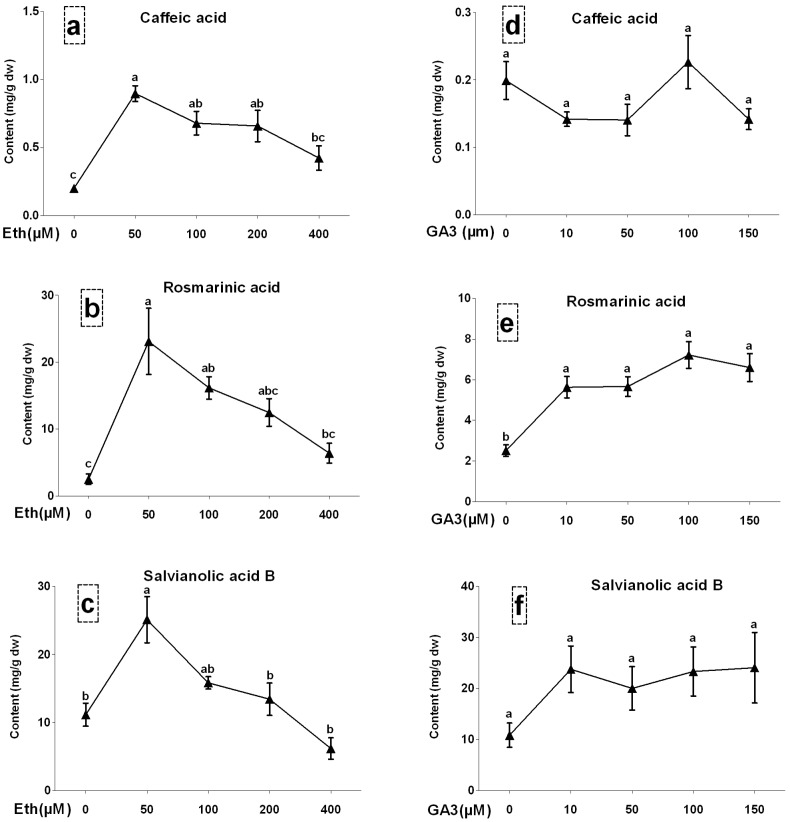
Effects of gibberellin acid and ethylene at different concentrations on production of salvianolic acid B, rosmarinic acid and caffeic acid in *S. miltiorrhiza* hairy roots. GA3, pibberellin acid 3; Eth, ethephon. Different letters indicate significant difference at *p*≤0.05 using Tukey test test. Means ± standard deviation (S.D.) (*n = 3*) are shown.

Three phenolic acids including caffeic acid (CA), rosmarinic acid (RA) and salvianolic acid B (SAB) in *S. miltiorrhiza* hairy roots were determined by HPLC. Effects of ABA at 50, 100, 150 and 200 µM on accumulations of three compounds have been reported in our previous work [8]. Effects of Eth at 50, 100, 200 and 400 µM on accumulations of three compounds were shown in Fig. 3abc. The CA and RA contents were significantly increased by 50 and 100 µM Eth treatments, and SAB content was significantly increased by 50 µM Eth treatment. The highest contents of three compounds reached to 0.90, 23.12 and 25.13 mg/g, respectively, in 50 µM Eth treatment. Fig. 3def showed effects of GA at 10, 50, 100, 150 µM on accumulations of three compounds. The results showed that CA content in the hairy roots was almost not affected by GA. Both RA and SAB accumulations were induced by GA, but differences of four treatments were not significant. Eth treatment was more efficient to enhance phenolic production than GA treatment. Responses of RA and SAB accumulations to three phytohormones were similar.

### Effects of Inhibitors on Phytohormone-induced Phenolic Production in *S. miltiorrhiza* Hairy Roots

To investigate further regulation mechanism of phytohormones on the biosynthesis of phenolic acids in *S. miltiorrhiza* hairy roots, inhibitors of phytohormone biosynthesis were applied into the hairy root cultures. Concentrations of ABA (50 µM), Eth (50 µM) and GA3 (100 µM) were selected according to the above work. Three phytohormones together with the related inhibitors were respectively added into the cultures.

**Figure 4:**
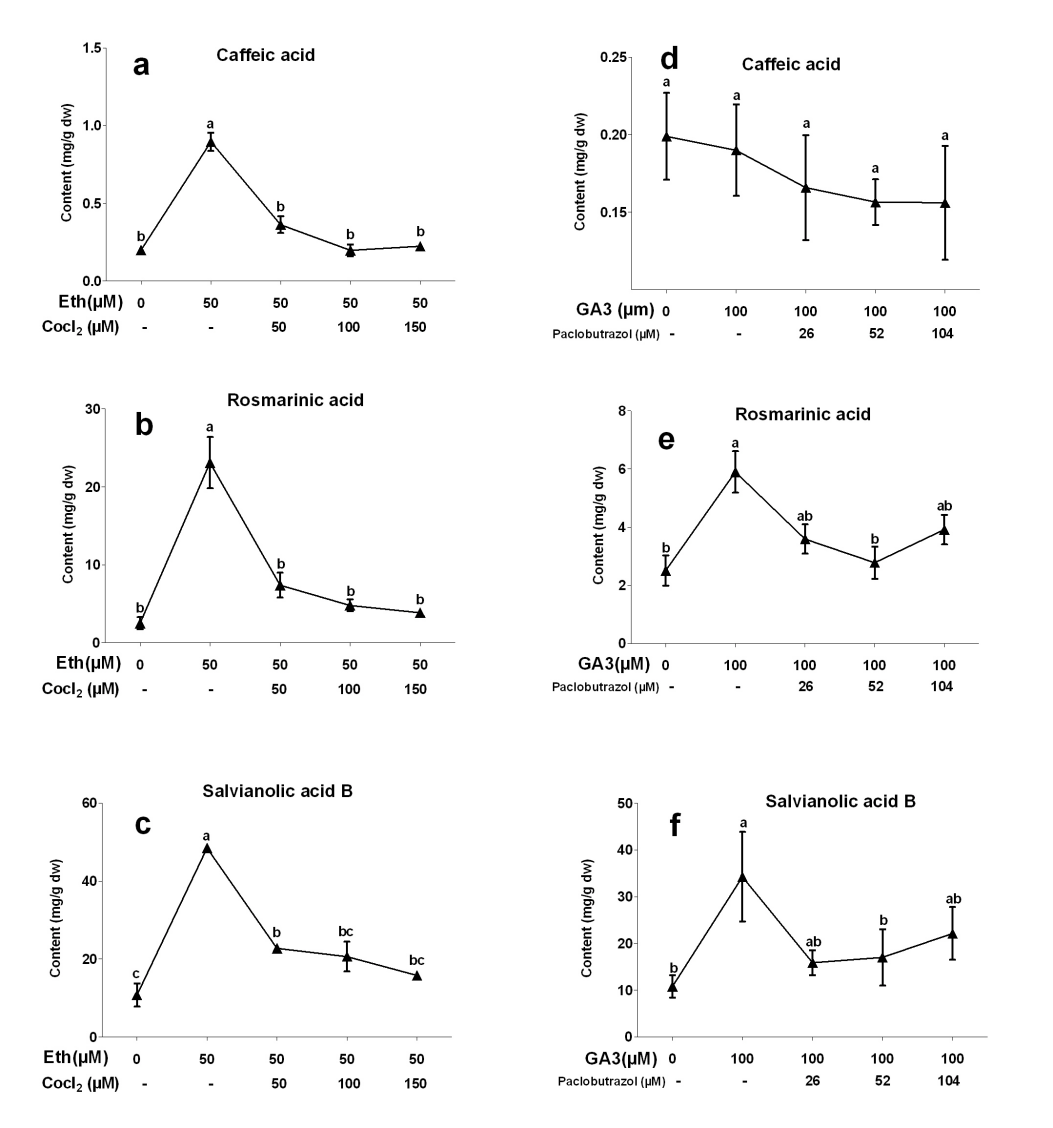
Effects of CoCl2 and paclobutrazol at different concentrations on production of salvianolic acid B, rosmarinic acid and caffeic acid in *S. miltiorrhiza* hairy roots. GA3, gibberellin acid 3; Eth, ethephon. Different letters indicate significant difference at *p*≤0.05 using Tukey test test. Means ± standard deviation (S.D.) (*n = 3*) are shown.

Fluridone was one of the efficient inhibitor of ABA biosynthesis. Effects of fluridone on ABA-induced phenolic production has been investigated in our work [8]. CoCl_2_ was an effective inhibitor of Eth biosynthesis in plants. In our experiments, three concentrations of CoCl_2_ (50, 100 and 150 µM) were separately applied into the cultures together with 50 µM Eth. The results showed that accumulations of three compounds induced by Eth were all significantly inhibited by CoCl_2_ treatment (Fig. 4a, b, c). The lowest contents of CA, RA and SAB were observed in 150 µM CoCl_2_ treatment. They were reduced by 75, 83 and 68% when compared to 50 µM Eth treatment. It suggested that phenolic production induced by the exogenous ethylene was probably related to endogenous ethylene. Paclobutrazol was an inhibitor of GA biosynthesis. Three concentrations of paclobutrazol (26, 52 and 104 µM) in combination with 100 µM GA3 were added into the cultures. The results showed that increases of RA and SAB contents induced by GA3 were significantly reversed by paclobutrazol (Fig. 4d, e, f). The lowest content of RA was observed in 52 µM paclobutrazol+100 µM GA3 treatment. It was decreased by 53% when compared to 100 µM GA3 treatment. The lowest SAB content was observed in 26 µM paclobutrazol+100 µM GA3 treatment, and was reduced by 54%. However, CA content was just slightly reduced by paclobutrazol treatment.

### Interaction between ABA and Gibberellin on Production of Phenolic Acids in *S. miltiorrhiza* Hairy Roots

**Figure 5:**
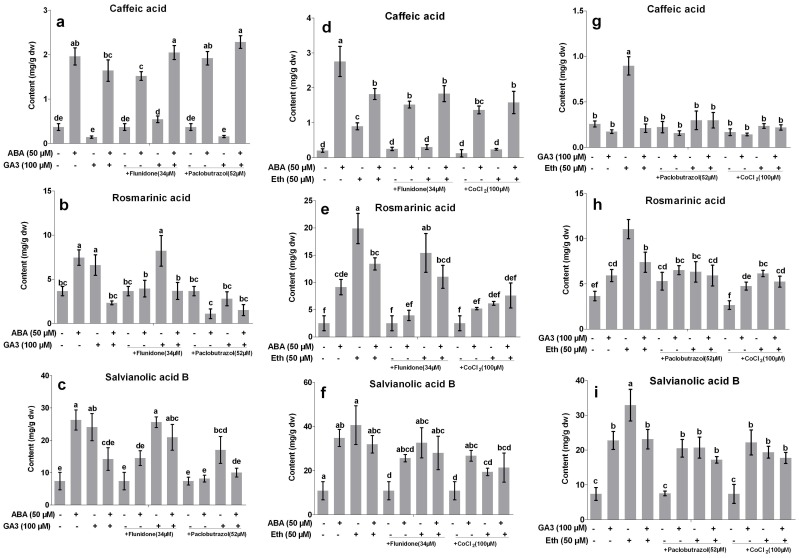
Interactions of abscisic acid, gibberellin acid and ethylene on production of salvianolic acid B, rosmarinic acid and caffeic acid in *S. miltiorrhiza* hairy roots. Eth, ethephon; GA3, gibberellin acid 3; ABA, abscisic acid. Different letters indicate significant difference at *p*≤0.05 using Tukey test. Means ± standard deviation (S.D.) (*n = 3*) are shown.

To elucidate interaction between ABA and gibberellin on production of phenolic acids, the combined effects of ABA, GA3, paclobutrazol and fluridone were investigated. Concentrations of 50 µM ABA, 100 µM GA3, 34 µM fluridone and 52 µM paclobutrazol were selected according to the above results. Fig. 5abc showed that contents of CA, RA and SAB were significantly increased from 0.37, 3.66 and 7.39 mg/g to 1.96, 7.45 and 26.3 mg/g by 50 µM ABA treatment. RA and SAB accumulation was also significantly increased to by 100 µM GA3 treatment. However, when ABA and GA3 were applied into the cultures together, RA contents were slightly reduced and SAB content was just slightly increased when compared to the control. Although CA content was significantly increased in ABA+GA3 treatment, the content was less than that in ABA treatment.

Treatments with paclobutrazol or fluridone alone almost could not affect contents of CA, RA and SAB in the hairy roots. The ABA-induced CA, RA and SAB production was significantly inhibited by 34 µM fluridone. Simultaneously, the ABA-induced RA and SAB accumulation was completely reversed by 52 µM paclobutrazol. Contents of RA and SAB in ABA+paclobutrazol were just 15% and 31% of the control levels. However, the ABA-induced CA production was almost not affected by paclobutrazol. The results indicated that endogenous GA was probably involved in ABA-induced RA and SAB production. Although increases of RA and SAB contents induced by GA3 were significantly reversed by paclobutrazol, they were almost not affected by fluridone. It suggested that endogenous GA was probably not involved in ABA-induced phenolic production in the hairy roots. Interestingly, CA production in ABA+GA3 treatment was significantly increased by paclobutrazol or fluridone.

### Interaction between ABA and Ethylene on Production of Phenolic Acids in *S. miltiorrhiza* Hairy Root

To study the interaction of ABA and ethylene on production of phenolic acids in *S. miltiorrhiza* hairy roots, the combined effects of 50 µM ABA, 50 µM Eth, 100 µM CoCl_2_ and 34 µM fluridone were investigated. Fig. 5def showed that CA content was increased to 13.8 folds of the control levels by ABA treatment and to 4.5 folds by Eth treatment. Although CA content was also significantly induced by ABA+Eth treatment, it was just 9.2 folds of the control levels. RA content in ABA and Eth treatments was increased from 2.5 mg/g to 9.1 and 19.9 mg/g, respectively. However, it was just 13.4 mg/g in ABA+Eth treatment. Significant increases of SAB content were observed in three treatments, but the differences of three treatments were insignificant.

Accumulations of three compounds were almost not affected by CoCl_2_ treatment alone. The ABA-induced CA production was significantly decreased from 2.8 mg/g to 1.4 mg/g by CoCl_2_ treatment, and increases of RA and SAB contents induced by ABA were slightly reduced by CoCl_2_ treatment. RA content in ABA+Eth treatment was significantly reduced by CoCl_2_ treatment, but CA and SAB production induced by ABA+Eth was almost not affected. The increases of three compounds contents in Eth treatment were significantly inhibited by fluridone.

### Interaction between Gibberellin and Ethylene on Production of Phenolic Acids in *S. miltiorrhiza* Hairy Roots

The combined effects of 50 µM Eth, 100 µM GA3, 100 µM CoCl_2_ and 52 µM paclobutrazol on production of phenolic acids in *S. miltiorrhiza* hairy roots were then investigated to reveal interaction between gibberellin and ethylene. Fig. 5ghi showed that contents of three compounds were largely improved by Eth. Although productions of RA and SAB were also induced by GA3, they were much less that those in Eth treatment. The combination of GA3 and Eth had no effect on CA content when compared to the control. RA and SAB contents were largely improved by GA3+Eth, but they were much less than those in Eth treatment. The Eth-induced phenolic production was significantly reversed by paclobutrazol treatment. Contents of CA, RA and SAB in Eth+paclobutrazol treatment were just 33%, 57% and 63% of the levels in Eth treatment. However, RA and SAB contents in Eth+GA3 treatment were just slightly reduced by paclobutrazol. Increases of RA and SAB contents in GA3 treatment were almost not affected by CoCl_2_, while those in GA3+Eth treatment were just slightly reversed by CoCl_2_.

### Effects of Phytohormones and Inhibitors on PAL and TAT Activities of *S. miltiorrhiza* Hairy Roots

PAL and TAT were two key enzymes involved the biosynthesis of phenolic acids in *S. miltiorrhiza* hairy roots. To reveal regulation mechanism of phenolic acids biosynthesis, effects of phytohormones and inhibitors on PAL and TAT activities of *S. miltiorrhiza* hairy roots were investigated.

**Figure 6:**
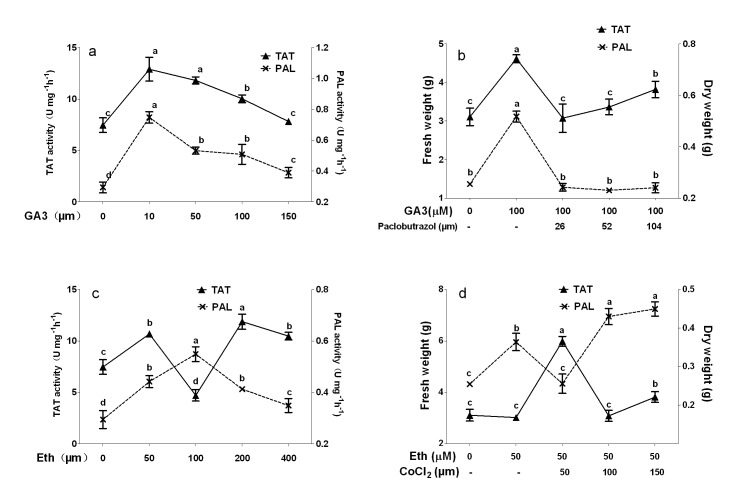
Effects of gibberellin acid, ethylene, paclobutrazol and CoCl_2_ on activities of PAL and TAT in *S. miltiorrhiza* hairy roots. GA3, gibberellin acid 3; Eth, ethephon; PAL, phenylalanine ammonia-lyase, TAT, tyrosine aminotransferase. Different letters indicate significant difference at *p*≤0.05 using Tukey test test. Means ± standard deviation (S.D.) (*n = 3*) are shown.

Effects of ABA and fluridone on PAL ad TAT activities of *S. miltiorrhiza* hairy roots have been reported in our previous work [8]. Responses of enzyme activities to GA and ethylene were shown in Fig. 6. The results showed that activities of PAL and TAT were significantly increased by GA3 treatments, and the highest values were observed in 10 µM GA3 treatment. Values of TAT and PAL activities in 10 µM GA3 treatment were increased by 154% and 73% when compared to the control. The GA3-induced TAT activity was completely reversed by 26 and 52 µM paclobutrazol treatments, and GA3-induced PAL activity was completely inhibited by paclobutrazol at three concentrations.

PAL activity was significantly improved by Eth at four treatments, and the highest value were observed in 50 µM Eth treatment (increased by 86% when compared to the control). TAT activity was significantly improved by 50, 150 and 200 µM Eth treatments and the highest value was observed in 150 µM Eth treatment (increased by 59% when compared to the control). However, TAT activity was significantly inhibited by 100 µM Eth, and the value was just 63% of the control level. The Eth-induced PAL activity was completely reversed by 50 µM Cocl_2_ treatment, while it was significantly enhanced by 100 and 150 µM Cocl_2_ treatment. Although TAT activity was not stimulated by 100 µM Eth treatment, it was significantly induced by 50 µM Cocl_2_+100 µM Eth treatment.

## Discussion

Various elicitors have been proved efficient to induce production of secondary metabolites in plant tissue cultures. Phytohormones, the special elicitors and plant growth regulators, have been widely used to stimulate accumulation of some important secondary metabolites in plants. Previous studies showed that accumulations of phenolic acids in plants were affected by ABA, GA3 and ethylene [12], [19]. ABA treatment could suppress PAL activity and phenolic acids accumulation in soybeans [19]. However, in strawberry, PAL activity and phenolic production were rapidly stimulated by ABA [12]. Our previous work suggested that activities of PAL and TAT as well as accumulations of CA, RA and SAB were significantly improved by ABA treatment [8]. It indicated that the responses of different plants to ABA were diverse and ABA was effective to stimulate the biosynthesis of phenolic acids in *S. miltiorrhiza* hairy roots. Ethylene was regarded as a plant senescence hormone, and it was effective to improve production of phenolic acids in strawberry [9] and carrots [10]. In *S. miltiorrhiza* hairy roots, application of ethephon led to significant increases of RA and LAB contents, but could not affect CA production. It was probably because that CA located on a branch pathway before RA and SAB biosynthesis, and ethephon facilitated metabolic flux to RA and SAB biosynthesis. Like ABA and ethylene, GA3 can also affect production of phenolic acids. Chlorogenic acid accumulation in tobacco callus was induced by high concentration of GA3, but was reduced by the low concentration treatment [20]. We found that CA production was not affected by GA3 treatment, while RA and SAB productions were significantly increased by GA3 at 10–150 µM. Simultaneously, PAL and TAT activities were significantly induced by Eth and GA3 treatments. It demonstrated that increases of phenolic acids contents in *S. miltiorrhiza* hairy roots induced by the three phytohormones were probably depended on the improvement of PAL and TAT activities.

Increasing evidence showed that effects of exogenous hormones on secondary metabolite accumulations can be arrested by their biosynthetic inhibitors. Our previous work showed that the ABA-induced tanshinone and phenolic productions were probably via the biosynthesis of endogenous ABA [5], [8]. Paclobutrazol was an inhibitor of GA biosynthesis. The GA3-induced tanshinone production were significantly inhibited by paclobutrazol in *S. miltiorrhiza* hairy roots [11]. We also observed that increases of RA and SAB contents induced by GA3 were significantly arrested by paclobutrazol. It was inferred that endogenous GA was probably involved in accumulations of RA and SAB under GA3 treatment. CoCl_2_ was considered to be effective to block the conversion of ACC to ethylene. Effects of ethylene on plants were significantly inhibited by CoCl_2_ [21]. In the present study, we found that CoCl_2_ could significantly reduce increases of phenolic production and enzyme activities induced by ethylene. The inhibitory effect of CoCl_2_ was probably caused by the inhibition of ethylene biosynthesis.

Interaction of phytohormones in regulating plant development and metabolism is a very complex process [9]. GA was regarded as an antagonist of ABA and ABA also acted as a competitive inhibitor of GA [15]. The antagonistic action between GA and ABA is an important factor regulating the developmental transition from embryogenesis to seed germination [14]. The same results were obtained in our experiments. Contents of phenolic acids in GA3+ABA treatment were less than those in GA3 or ABA treatment alone. It demonstrated that the antagonistic action between ABA and GA played an important role in phenolic production. ABA acted through inhibition of GA biosynthesis [22]. We obtained the same results. The increase of RA and SAB induced by ABA was significantly inhibited by paclobutrazol, which suggested that ABA-induced phenolic production probably depended on GA signaling. Yet, GA3-induced phenolic production was almost not affected by the inhibitor of ABA biosynthesis.

It has been suggested that effect of ethylene could be counteracted directly by the application of ABA [16]. In the opposite, effect of ABA could be also counteracted by ethylene [17]. Enhanced levels of ethylene are known to reduce the endogenous ABA concentration in rehydrated *Xanthium* leaves [23]. ABA can antagonize ethylene-induced hyponastic growth in Arabidopsis [24]. In *S. miltiorrhiza* hairy roots, we found that ABA-induced CA production was counteracted by ethylene, and ethylene-induced RA production was counteracted by ABA. It demonstrated that the ABA-ethylene antagonistic action was involved in production of phenolic acids in *S. miltiorrhiza* hairy roots. The previous work suggested that the function of ABA signaling in the root growth requires the cascade of ethylene signaling [25]. ABA treatment led to increase of ethylene production, and application of inhibitors of ethylene biosynthesis reversed the inhibitory effect of ABA on flowering [26]. The inhibitory effect of ethylene on *Pharbitisnil* flowering may depend on its influence on the ABA level [26]. In tomato, exogenous ABA treatment promoted ethylene synthesis and fruit ripening, and it was proposed that ABA may act at upstream metabolic events of ethylene action/perception [27]. In our experiments, we observed that ABA-induced phenolic production was reversed by CoCl_2_ and ethylene-induced phenolic production was also inhibited by fluridone. It was suggested that ethylene was probably involved in ABA-induced phenolic production and ABA probably also participated in ethylene-induced response of *S. miltiorrhiza* hairy roots. It was inferred that the balance between ABA and ethylene levels probably played an important role in the biosynthesis of phenolic acids.

Ethylene and gibberellic acid are antagonistic on growth of seedlings of *Alaska pea*. Ethylene interfered severely with the action of gibberellic acid but did not completely suppress it [18]. In the other hand, the potential of GA3 to counteract the effects of ethylene has also been reported [28]. Although both GA3 and ethylene could increase RA and SAB content, the ethylene-induced effects were antagonized by GA3 treatment. Notably, the increase of CA content in ethylene treatment was completely suppressed by GA3. It indicated that GA3 acted as a competitive inhibitor of ethylene on the biosynthesis of phenolic acids. However, in deepwater rice, ethylene and GA have a synergistic effect on the growth rate of emerged roots [15]. Ethylene-mediated enhancement of apical hook formation in *Arabidopsis thaliana* seedlings is gibberellin dependent [29]. Our results showed that ethylene-induced phenolic production was also inhibited by paclobutrazol, which suggested that GA signanling was probably involved in the ethylene-induced biosynthesis of phenolic acids. In lowland rice, gibberellin-dependent early response to submergence is not necessarily mediated by ethylene [30]. This conclusion held true for *S. miltiorrhiza*. We observed that effects of GA3 on phenolic acid accumulation was not affected CoCl_2_. Probably, ethylene signaling is not necessary for GA3-induced phenolic production.

In conclusion, phytohornones including ABA, GA3 and Ethylene were effective to improve production of phenolic acids and increase activitives of PAL and TAT in *S. miltiorrhiza* hairy roots. Antagonistic actions between the three phytohormones played important roles in the biosynthesis of phenolic acids. GA signaling is necessary for ABA and ethylene-induced phenolic production. Yet, ABA and ethylene signaling is not necessary for GA3-induced phenolic production. The complex interactions among phytohormones help us understand regulation mechanism of secondary metabolism and scale-up production of active ingredients in plants.
